# Social and economic dimensions of food sustainability – a background paper for the Nordic Nutrition Recommendations

**DOI:** 10.29219/fnr.v68.10450

**Published:** 2024-02-05

**Authors:** Peter Jackson, Lotte Holm

**Affiliations:** 1 Institute for Sustainable Food, University of Sheffield, Sheffield, UK; 2 Department of Food and Resource Economics (IFRO), University of Copenhagen, Copenhagen, Denmark

**Keywords:** sustainable food systems, dietary guidelines, food systems, socioeconomic dimensions

## Abstract

This review seeks to demonstrate how the social and economic dimensions of sustainability need to be considered alongside its environmental dimensions. This is particularly important when, as in the case of the Nordic Nutrition Recommendations (NNRs), policymakers are attempting to address the twin goals of health and sustainability. For a policy that might make good sense when seen in purely environmental terms, it might not prove sustainable in social and economic terms – if it is too costly, it exacerbates existing inequalities or has detrimental effects on public health. There are some ‘win-wins’ in the field of health and sustainability policy. However, difficult choices between competing policy options will occur, similar to those facing ordinary consumers in their everyday lives. Being clear about the way food is framed as an issue and how different framings shape policy outcomes is a useful way forward in addressing the inevitable trade-offs and compromises between competing objectives.

## Popular scientific summary

- This review discusses the social and economic sustainability dimensions of food consumption.
- Whilst there are potential ‘win-wins’ in the field of health and sustainability policy, trade-offs and compromises between competing policy objectives are inevitable.
- This is particularly so when taking the social and economic dimensions of sustainability into account.
- Being transparent about how the food system is framed is a useful way forward in addressing competing policy options.

The current revisions to the Nordic Nutrition Recommendations (NNRs) (1) attempt to address the health effects of foods as well as the environmental sustainability challenges associated with dietary change. In this, they are following the Food and Agriculture Organization (FAO) guiding principles on healthy sustainable diets (see Box 1).

Box 1 Sustainable healthy diets

Sustainable Healthy Diets are dietary patterns that promote all dimensions of individuals’ health and well-being; have low environmental pressure and impact; are accessible, affordable, safe and equitable; and are culturally acceptable. The aims of Sustainable Healthy Diets are to achieve optimal growth and development of all individuals and support functioning and physical, mental and social well-being at all life stages for present and future generations; contribute to preventing all forms of malnutrition (i.e. undernutrition, micronutrient deficiency, overweight and obesity); reduce the risk of diet-related Non-communicable diseases (NCDs); and support the preservation of biodiversity and planetary health. Sustainable healthy diets must combine all the dimensions of sustainability to avoid unintended consequences (4).

This paper reviews the scientific evidence on the social and economic dimensions of food sustainability particularly insofar as the evidence relates to the Nordic and Baltic countries. It is a background paper to inform the proposed revisions to the NNR and does not offer specific recommendations (Box 2).

Box 2 Background papers for Nordic Nutrition Recommendations 2023

- This paper is one of many scoping reviews commissioned as part of the NNR 2023.
- The papers are included in the extended NNR2023 report, but, for transparency, these scoping reviews are also published in *Food & Nutrition Research.*
- The scoping reviews have been peer reviewed by independent experts in the research field according to the standard procedures of the journal.
- The scoping reviews have also been subjected to public consultations (see report to be published by the NNR2023 project).
- The NNR2023 committee has served as the editorial board.
- Whilst these papers are a main fundament, the NNR2023 committee has the sole responsibility for setting dietary reference values in the NNR2023 project.

## Sustainability’s multiple dimensions

Sustainability is usually defined in terms of the impact of current practices on the prospects of future generations, a definition that goes back to the UN Brundtland Report, which defined sustainability as ‘meeting the needs of the present without compromising the ability of future generations to meet their own needs’ (2). Incorporating the concept of ‘needs’, the Brundtland definition is both an environmental and a people-centred (social) definition. It is widely acknowledged that sustainability has multiple dimensions, including ecological (sometimes called environmental), social and economic aspects. Indeed, when the UN General Assembly adopted the 2030 Agenda for Sustainable Development (from which the 17 Sustainable Development Goals were derived), they committed the global community to ‘achieving sustainable development in its three dimensions – economic, social and environmental – in a balanced and integrated manner’ (3). There has since been widespread debate about how many dimensions sustainable development has, with some authors proposing several additions – technical, legal, political and moral – besides the original three (5).

But what does this mean in practice? For a policy or intervention to be truly sustainable, it must meet a range of criteria. It would not be sustainable if it were environmentally sound, having a neutral or negative impact on CO_2_ emissions, for example, if it was not possible to adopt the policy or practice without increasing social inequalities or if it was economically impossible to maintain in future because it was prohibitively expensive or led to increased (and ultimately unsustainable) economic disparities. This formulation is similar to Halkier’s argument that for an innovation to succeed, as well as being economically viable, it must also be technically feasible and culturally appropriate (6). Failure to meet one or more of these criteria will render any innovation unsustainable in the longer term even if it appears to be successful in the shorter term.

The remainder of this review seeks to outline some of social and economic dimensions of sustainability, based on evidence available in the peer-reviewed literature. In writing this paper, we have not undertaken our own systematic review of the scientific evidence. Rather, we have relied on a recent Evidence Review Report undertaken by an expert group of academics, convened under the auspices of Scientific Advice for Policy by European Academies (SAPEA), of which we were both members. The SAPEA report provides an independent review of the evidence required to inform the transition to a more just and sustainable food system for the EU, including the identification of ‘a practice’ examples, some of which are drawn from the Nordic and Baltic countries (see below) (7).

This paper proceeds as follows. The next section provides a summary of the SAPEA report, drawing out its implications for healthy and environmentally sustainable diets. The limitations of the report are summarised, including a comment on recent events such as the impact of the COVID-19 pandemic, the war in Ukraine and the current cost-of-living crisis affecting many Western economies. This paper then outlines some of the social and economic dimensions of sustainability, including inequalities of access and affordability, questions of cultural appropriateness and power asymmetries within existing food systems. It covers sustainability issues related to production and consumption, acknowledging that the balance between the environmental, social and economic dimensions of sustainability is not well understood. This review also provides a commentary on trade-offs and compromises between competing objectives and some reflections on the factors that may limit the durability of initiatives that are designed to increase the health and sustainability of contemporary food systems.

## The SAPEA report

Funded by Horizon Europe as part of the European Commission’s Science Advice Mechanism, the SAPEA working group was asked to consider how a socially just and sustainable food system for the EU is best defined and described, based on the available scientific evidence and covering the societal, economic and environmental dimensions of sustainability. The remit was extremely wide including the identification of workable paths to deliver an inclusive, just and timely transition to an EU sustainable food system, seeking ‘co-benefits’ for health and environment and taking account of the socio-economic situation of the farming sector, a range of territorial imbalances, the rural-urban divide, the nature of food waste and the responsibilities of consumers and other food system actors. The SAPEA report focused on policies and practices whose efficacy has been scrutinised in the peer-reviewed academic literature, attempting to identify ‘what works’ in terms of policy and practice, including the barriers and enablers of change towards a more sustainable and socially just food system.

The working group’s report was subject to review by external experts and a wide range of stakeholders, including those with practical knowledge and experience. It was then considered by the Group of Chief Scientific Advisors (GCSA) who produced a Scientific Opinion including a series of recommendations to the European Commission. The process included a series of checks and balances, designed to ensure that its advice was impartial and evidence-based, free from bias and political lobbying.

As well as writing an Evidence Review Report, the working group also oversaw the production of a series of systematic literature reviews including an overview of the policy landscape, a review of definitions and theoretical perspectives and a review of ‘good practice’ examples. All of these reviews, together with the Evidence Review Report and the GCSA’s Scientific Opinion, are now in the public domain (https://sapea.info/topic/food/).

The following paragraphs provide a summary of the SAPEA report, emphasising those sections that are most relevant to the NNR.

The Report began with a discussion of alternative definitions of ‘sustainable food’, concluding that the High Level Panel of Experts on Food Security and Nutrition (HLPE) definition was a good starting point, defining a sustainable food system as one ‘that ensures food security and nutrition for all in such a way that the economic, social and environmental bases to generate food security and nutrition of future generations are not compromised’ (8). These definitions are highly contested. For example, Béné et al. suggest that definitions of sustainability reflect distinctive disciplinary narratives with different epistemological assumptions, mental models and disciplinary paradigms (9). They conclude that, although the concept is widely used by diverse communities of practice, it remains poorly defined and applied in diverse ways. The authors also assert that trade-offs between different dimensions of food system sustainability are unavoidable and need to be addressed explicitly when implementing sustainability initiatives.

Based on the evidence reviewed, the SAPEA report concluded that sustainability and food security are amongst the greatest challenges facing the world today. Referring to the idea of planetary limits and the ‘safe operating space for humanity’, Rockström et al. showed that the three areas where we are already operating well beyond these limits are intimately connected to food (10). They are biodiversity (where intensive agriculture poses a significant threat, reducing the diversity of habitats on which wildlife flourishes), the nutrient cycle (where the widespread application of pesticides, fungicides and fertilisers causes significant disruption) and climate change (where the food system is estimated to contribute around one-third of global greenhouse gas emissions). The Royal Society report on ‘Nourishing 10 billion sustainably’ provides a useful compilation of evidence on these issues (11).

Adopting a systems approach helps recognise synergies and trade-offs, moving beyond linear ‘farm to fork’ approaches to more circular, inclusive systems (12). Ericksen suggests that it is relevant to analyse how food systems interact with global environmental change and to evaluate ‘the major societal outcomes affected by these interactions: food security, ecosystem services, and social welfare’ (p. 235) (13). A systems approach helps make connections across the food system including waste reduction and the stimulation of healthier diets. It also facilitates the recognition of power asymmetries, complex governance arrangements and regulatory challenges.

The SAPEA report reviewed a range of potential public health interventions, including the so-called ‘hard’ instruments, such as taxes, certification and standards; outright bans; and ‘softer’ measures that provide information and advice or attempt to ‘nudge’ consumers to make healthier or more sustainable decisions by adjusting the ‘choice architecture’. These terms are derived from behavioural economics including the influential work of Thaler and Sunstein (14). Experimental approaches, where innovations are trialled and evaluated, often at the local level, can be powerful in this context, offering a means of identifying specific leverage points within a complex system, allowing adjustments to be made and conflicts to be addressed (see, for example, Watson’s work on change points) (15).

Recent initiatives across Europe provide examples where actors, issues and contexts of transformation have been successfully coordinated. These include taxation schemes, producer and consumer cooperatives, technological initiatives, labelling and governance initiatives, socio-economic initiatives, health and sustainability initiatives, and multilevel collaborations to promote sustainable food cities. Specific examples from the Nordic and Baltic countries are listed as follows.

The SAPEA report concluded that fundamental, system-wide changes were required in order to promote the transition towards a fairer, more sustainable and healthier food system. Environmental, health and socio-economic issues are thoroughly interconnected and do not exist in separate silos.

Meeting the growing global demand for food will require significant dietary change as well as large reductions in food waste, as technological change or yield increases are unlikely to meet demand alone. Evidence of ‘what works’ in policy terms requires strengthening, including further research on the public understanding of science and consumer acceptance of new technologies.

The SAPEA report noted that whilst food systems are increasingly globalised, significant variations exist within and between EU states. Sustainability policies will therefore need to address the diversity of national economies (including the significance of the farming sector) and territorial imbalances between urban and rural areas. This variation also applies within and between the Nordic and Baltic states (see Table 1). For example, income (Gross Domestic Product [GDP] per capita) is 2–3 times higher in the Nordic countries than in the Baltic states, and the Nordics are amongst the 20 richest countries in the world, whilst the Baltic countries are ranked between 44 and 53. This also means that food accounts for a much larger share of expenditure on consumption in the Baltic states, almost twice the level in the Nordic countries.

**Table 1:** Key figures for the agricultural and food situation in the Nordic and Baltic countries (2021 or latest year with available data)

		Denmark	Norway	Sweden	Finland	Iceland	Estonia	Latvia	Lithuania
Food supply: vegetal products	kcaVcapita/day	2.177	2.349	2.136	2.043	1.974	2.061	2.289	2.450
Food supply: animal products	kcaVcapita/day	1.244	1.100	1.048	1.277	1.663	1.106	964	960
Food supply: Fish, Seafood	kcaVcapita/day	75	103	58	62	187	22	56	68
Food supply: Milk - Exdudng Butter	kcaVcapita/day	472	326	441	491	623	572	342	499
Food supply: Meat, total	kcaVcapita/day	304	386	300	469	554	317	310	435
Food’s share of total consumption	Pct	11	12	13	12	18	21	19	23
Rural population, share of total	Percent	11,7	17,0	11.4	14,6	5,7	29,9	31,6	32,4
Agriculture, forestry, and fisting, value added	Per cent of GDP	1.0	1.6	1.3	2,4	na	2,1	4,0	3,3
Agricultural raw materials exports	Pct of merch. export	2,3	0,6	4,8	8.4	0,6	7.9	12,9	3,1
Food exports	Pct of merch. export	17,2	9.3	6,3	2,5	46,0	9,1	18,0	16,5
GDP per capita (2020)	USD	61.063	67.330	5Z300	49.161	59.264	23.054	17.704	20.232
GDP per capita (2020), rank	Number	9	7	15	18	12	44	53	48
Arable land per holding	Hectare	60	19	38	41	47	36	16	13
Number of dairy cows per holding	Dairy cows	180	31	85	35	na	49	9	5
Self sufficiency rate - pigmeat	Prod/consunp.*100	750	100	81	93	100	87	50	56
Set sufficiency rate - vegetables	Prod/consunp.*100	44	45	34	52	19	23	35	62
Set sufficiency rate - fish	Prod/consunp.*100	190	210	70	80	1150	380	165	100
Net export (export-import), agricultural prod.	Million USD	5.152	-6.648	-5.922	-3.658	-563	-220	102	1.936
Net export (export-import food excl fish	Million USD	3.043	-7 065	-6821	-4071	-593	-265	-145	353

*Source*: FAO and Eurostat. We are grateful to Dr Henning Otte Hansen from the Department of Food and Resource Economics at the University of Copenhagen for compiling these data.

Linked to income differences and to a less extent price differences, the pattern of food supply varies considerably from country to country. The Baltic countries have a relatively low supply of animal products and a relatively high supply of vegetable products. The supply of fish is also very different from country to country, reflecting the extent of domestic production.

Agricultural and rural population accounts for around 30% of the population in the Baltic countries, double the level in the Nordics. The structure of agriculture – and thus also to a certain extent the level of international competitiveness of agriculture and future production potential – varies greatly from country to country. In general, agriculture in the Baltics is characterised by relatively small units – for example, on average only 5–9 dairy cows per dairy cow farm in Latvia and Lithuania, compared to 180 in Denmark.

The Nordic and the Baltic countries together are net importers of both agricultural products and food (excluding fish). However, there are large differences amongst the countries, as Denmark and Lithuania are large net exporting countries.

A ‘one size fits all’ policy on nutrition and sustainability is clearly inappropriate in these circumstances where due attention needs to be paid to national and local variation. Dietary guidelines, such as the NNR, should be flexible and adapted to local circumstances rather than uniform or overly prescriptive.

The SAPEA report identified a series of ‘good practice’ examples where there was strong peer-reviewed evidence of positive long-term impacts, including health and sustainability benefits. Examples included state support for the growth of the Danish organic sector (16); the RETHINK project in Latvia and Lithuania – an action-research programme that explored the structures and opportunities for small and medium-size agricultural holdings that are not well incorporated into the mainstream market (17); and the Danish Wholegrain Partnership that achieved a significant increase in wholegrain consumption through a process of multi-sector collaboration involving the Danish Veterinary and Food Administration, the food industry and health NGOs such as the Danish Cancer Society (18, 19).

The SAPEA report also noted a series of other initiatives, including the Finnish Nutrition Commitment (https://www.ruokavirasto.fi/en/foodstuffs/healthy-diet/nutrition-commitment/), which encourages food business operators and stakeholders to improve the nutritional quality of the national diet and to adopt nutritionally responsible practices; the ForMat project in Norway (https://norsus.no/wp-content/uploads/or1716-format-sluttrapport-english.pdf), which aimed to achieve a significant (25%) reduction in edible food waste; the Danish salt partnership (https://altomkost.dk/fakta/kort-om-naeringsstoffer/salt/), which aimed to reduce the intake of salt amongst consumers through increased awareness of the link between salt and health as well as collaboration with the food industry on reducing the salt content in processed food; and the Norwegian Partnership for a Healthier Diet (https://www.helsedirektoratet.no/english/partnership-for-a-healthier-diet), which aims to increase the proportion of the population who have a balanced diet in accordance with the official dietary guidelines. The partnership contains specific goals related to reducing the intake of salt, added sugar and saturated fat, and increasing the intake of fruits and berries, vegetables, whole grain foods, and fish and seafood in the population. There is also a goal of increasing the sale of foods labelled with the Keyhole label. The goals are in line with the challenges in the Norwegian diet, as outlined in the Norwegian National Action Plan for a Healthier Diet (Norwegian National Action Plan for a Healthier Diet, regjeringen.no). The partnership was established in 2016 and will continue to the end of 2025 (Helsedirektoratet. Partnership for a healthier diet: https://www.helsedirektoratet.no/english/partnership-for-a-healthier-diet).

Other initiatives from the Nordic and Baltic states that were noted in the SAPEA report include Matsentralen (https://www.matsentralen.no/), a non-profit organisation that fights food waste and helps disadvantaged people by redistributing surplus food at risk of going to waste; SkolmatSverige (https://www.skolmatsverige.se/), which supports Swedish primary schools in their work to provide good school meals; and Eldrimner (https://www.eldrimner.com/), which provides knowledge, support and inspiration to artisanal food producers throughout Sweden and the Nordic region, including those at the early stages of their careers. We also note several other relevant initiatives, besides those mentioned in the SAPEA report, including the Danish Council for Healthier Food (Rådet for sund mad (raadetforsundmad.dk), along with the Food partnership for health and climate (foedevarestyrelsen.dk) and the Stop Food waste organisation (Stop Madspild).

Finally, the SAPEA report noted that sustainability transitions raised significant governance challenges. Food system governance often transcends the boundaries of individual nation-states, crossing different policy domains (environment, trade, food safety, etc.) and including private standards and certification schemes as well as formal regulatory systems. There are also significant variations in terms of the EU’s competencies to address different parts of the food system. For example, the EU has strong competencies in terms of agriculture and fisheries but more limited influence over public health, which remains largely within national governments’ legal competencies. Recognising the multi-layered nature of food system governance, within and between nations, is a necessary first step in promoting a more coordinated and integrated approach. Food system governance is also rendered challenging in terms of the number and diversity of non-governmental agents involved, including food producers in farming and fishing; manufacturers, processors and packaging experts; those involved in transportation and distribution; retailers; educators; consumers; NGOs and civil society organisations; scientists and researchers. The SAPEA report concluded that good food system governance requires system-based problem framing, boundary-spanning structures, adaptability, inclusiveness and transformative capacity. The use of science and technology in future food system governance also raises important challenges in terms of public engagement, consumer acceptance and trust, as recent debates over biotechnology have shown.

## Limitations of the SAPEA report

The SAPEA report is not without its limitations. It was completed in April 2020, immediately prior to the outbreak of the COVID-19 pandemic in Europe. The pandemic had numerous impacts on the food system that did not figure in the SAPEA report beyond some general discussion of unpredictable events, future scenarios and food system resilience. The pandemic served to highlight the potential vulnerability of just-in-time food supply chains, leaving temporary gaps on supermarket shelves and leading to accusations of ‘panic buying’ amongst consumers. See, for example, Ritzel et al.’s interpretation of the motivation for ‘excessive’ food buying (20), and Benke’s alternative interpretation of stockpiling as a rational form of ‘resistance’ to the uncertainties of lockdown (21). Food services such as pubs, cafes and restaurants were subject to temporary closure and other restrictions, colloquially referred to as ‘lockdown’, whilst online shopping for food and other goods underwent a surge in popularity. In many countries, the pandemic increased existing social inequalities, pushing many more people into food poverty and ‘normalising’ the provision of emergency food aid (22). Rivera Ferre et al. have also argued that, through land use changes and habitat fragmentation, industrialised food systems are a driver of infectious diseases such as COVID-19, and that the recent pandemic may serve as a prompt for food system change (23).

Other recent events have had a significant effect on the sustainability of European food systems, with adverse effects on the most vulnerable in society. Besides its devastating effects in Ukraine itself, the war in Ukraine has led to escalating energy costs, disrupted food exports, increased labour shortages and uncertain future harvests, exacerbated by a shortage of fertilisers (24). Others have argued that the Russian invasion of Ukraine has created new food insecurities whilst highlighting existing systemic weaknesses in international food security (25).

Beyond Ukraine, the current cost-of-living crisis and rising inflation across Europe have had further negative consequences for the sustainability of food systems, with severe consequences for public health, leaving many households facing difficult choices about ‘heating or eating’ (26). Whilst the onset of specific events may be hard to predict, the resilience of food systems to future ‘shocks’ – environmental, political and economic – should be a major focus of future research where scenario analysis offers a potentially valuable way forward (27).

## Social and economic dimensions of food sustainability

Most accounts of the relationship between dietary change and sustainability in the Nordic countries pay more attention to environmental sustainability than to its social and economic aspects (and few have considered the *weighting* that should be accorded to the difference dimensions) (28). Here, we focus on the social and economic dimensions of food sustainability.

Social sustainability is a multidimensional concept, with no universal definition. Core themes concern human well-being, equity and fairness, equality of rights, access to basic needs, justice, social inclusion and participation – and more (29). Furthermore, it has been suggested that the key dimension to social sustainability is maintenance or preservation of sociocultural characteristics in the face of change, and the ways in which people actively embrace or resist those changes (30).

Emphasising the social and economic dimensions of sustainability should begin by acknowledging the implications of different ‘framings’ of food. Whether food is seen as a commodity, a human right or a common good leads to very different policy formulations. For example, in policies supporting business innovation, product differentiation and ‘nudging’ to change consumer behaviour, food is seen as a commodity. In policies supporting vulnerable consumer groups, such as public procurement initiatives and interventions to improve the quality of school meals, food is seen as a human right, whilst in supporting civil society participation and rural-urban food coalitions, food is seen as a common good (31). Similarly, framing the issue in terms of food security and sustainability or in terms of food justice, equity and sovereignty is much more than a semantic choice. It is therefore important that these framings are made explicit in debates about policy and practice relating to food.

The next two sections focus on the sustainability of food production and consumption. Whilst they are presented in separate sections, a ‘food systems’ approach (as advocated in the SAPEA report) insists on their inseparability. With this in mind, we also include a discussion of the trade-offs and compromises between different aspects of sustainability as they cross-cut these two spheres.

### The sustainability of food production

Whilst much of this review focuses on the sustainability of *consumption* practices, we also recognise the need for *production* systems to become more socially and economically sustainable. This includes the sustainability of the farming sector where dietary change may have important implications for food security, national self-sufficiency and employment across the Nordic countries and Baltic states. In their review of the challenges of sustainability for agriculture and food economics, for example, Brunori et al. conclude that, as well as consumption patterns becoming more aligned with healthy and sustainable diets, production systems should reduce their pressure on natural resources, food supply systems should be made more resilient, and effective enforcement, monitoring and evaluation systems for food-related policies should be developed (32).

Whilst there have been some studies of the impact of climate change on Nordic agriculture including policies in support of environmental sustainability and green growth (33), less attention has been paid to the social and economic sustainability of the agri-food sector with very little research at the farmer level (34). Amongst the exceptions to these trends, Huan-Niemi et al. discuss concerns throughout the Nordic countries at the prospect of a significant decrease in the consumption of animal-based foods (35). In Finland, for example, they report that meat and dairy products account for almost 32% of the average household’s food purchases (36), whilst livestock production represents 47% of the market turnover for primary agricultural production (37). There are also concerns about national self-sufficiency, with around 50% of the fruits and vegetables purchased in Finland currently imported (38). A transition towards more plant-based diets would therefore require significant investment to maintain the prosperity of the agri-food industry, including increased investment in legume production and processing. Shifts to a more plant-based diet also raise concerns about food security and employment in the agri-food sector (a point that was raised by several commentators during the open consultation process on an earlier draft of this paper). The shift to plant-based diets is moving particularly rapidly in Denmark, where sales of plant-based dairy alternatives increased from 72 to 277 million dkr from 2014 to 2019 (https://www.euromonitor.com/drinking-milk-products-in-denmark/report).

In considering the social and economic dimensions of sustainable dietary change in the Nordic countries and Baltic states, it is important to recognise the extent of national differences in primary food production including crop and livestock production and differences in export orientation. For example, as Meltzer et al. contend, Denmark is a major exporter of dairy products (such as butter) and meat (mainly pork), Norway is the world’s largest salmon exporter, Iceland exports seafood and Sweden exports fish, whilst dairy is the biggest food export of Finland (28). These national differences need to be born in mind when considering the likely impact of dietary change on each country’s agri-food sector. In their discussion of the Nordic diet, for example, Karlsson et al. include a short discussion of the consequences for farmers of a reduction in livestock farming and/or an increase in organic production (39). Considering the likely impact of a shift towards increased production of vegetables and pulses, they point to the need for new policy instruments to ensure the economic sustainability of farmers. They also note its potential impact on existing infrastructure such as dairies and slaughterhouses and highlight the scope for a revival of the cooperative movement.

Huan-Niemi et al. also report regional differences in the production of protein-rich foods (35). For example, whilst Southern Finland has some potential for increasing pulse production, elsewhere in Finland, the replacement of livestock production with more diverse plant production will be significantly more difficult. They also suggest that a significant drop in the demand for meat and dairy products may cause considerable economic and social problems at a local level, including decreased employment and tax revenues in rural areas.

The economic dimensions of food sustainability also include issues of land ownership, food production and corporate power. For example, the EU’s Farm to Fork strategy sought to preserve the affordability of food whilst generating fairer economic returns to food producers, fostering the competitiveness of the EU supply chain and promoting fair trade (40). It recognised that a transition to a more sustainable food system will lead to changes in the economic fabric of many EU regions and their patterns of interaction, including how SMEs are affected and how innovation is fostered. The Farm to Fork strategy also sought to adopt greener business models, encouraging the adoption of a circular bio-based economy and a shift to more renewable forms of energy.

### Sustainable consumption practices

As well as acknowledging the environmental implications of changing production practices, as discussed by Meltzer et al. in relation to the Nordic diet, addressing the social and economic dimensions of sustainability also needs to address the significance of changing consumption practices (28). Here, it is important to acknowledge that consumption practices are deeply embedded within relational, institutional and cultural contexts (41). This implies that dietary change involves processes of adaptation, on many levels and by many actors, and that such adaptations evolve from already specific social and cultural configurations. To promote the social sustainability of dietary change, that is to embed changes into everyday life in equitable and durable ways, food consumption practices should be seen to be affected by social norms and conventions, social relations, institutional arrangements, and the organisation of daily life in time and space. Within the Nordic countries, for example, significant and stable variations in meal patterns are found between Denmark and Norway’s cold lunches and Finland and Sweden’s hot lunches, likely reflecting different institutional arrangements of meals in schools and workplaces (42), but also suggesting variations in social contexts of lunches: More people eat lunch alone in Denmark and Norway than in Sweden and Finland (43). However, overall, for most people in the Nordic countries, most meals are shared with family members, friends, colleagues and others (44), and gathering all household members for shared meals happens frequently and regularly in all Nordic countries (45). It follows that changing diet is not only a decision that individuals make autonomously and solely on the basis of personal preferences but also a process that requires negotiations with and adaptations to the wants and needs of significant others (46). Shared cultural norms and conventions about what is a ‘proper meal’ (47) and what is a good and appropriate food are therefore pivotal points to take into consideration when recommending dietary change.

Addressing the social dimensions of food sustainability also requires a sensitivity to inequalities of access, affordability and price which are often structured by class, ethnicity and gender and their complex intersectionalities. These issues are reflected in standard definitions of food security, which acknowledge the importance of *social and economic access* as well as physical access to food, making reference to *safe and nutritious* as well as sufficient food and accepting the importance of food that meets people’s *dietary needs and food preferences*. These issues are all included in the standard definition of food security, which is said to exist when ‘all people, at all times, have physical, social and economic access to sufficient, safe and nutritious food to meet their dietary needs and food preferences for an active and heathy life’ (48). They are even more prominent in alternative rights-based formulations such as those that focus on food poverty, defined in terms of the inability to acquire or eat an adequate quality or sufficient quantity of food in socially acceptable ways (or the uncertainty of being able to do so) (49). The emphasis here is on the debilitating effects of future uncertainties about access to food as well as on current shortages, and the reference to ‘socially acceptable ways’ is intended to challenge the long-term reliance on stigmatising forms of emergency food aid such as foodbanks.

Attempts to provide nutritional advice, based on the optimisation of dietary intake, need to take account of social differences and cultural variations amongst consumers. For example, the consumption of fresh vegetables and fruit may be desirable in terms of their nutrient content, but to consumers facing economic hardship, they may be seen as unaffordable because of cost or risk of waste (50).

Considering the impact of dietary change on a global scale, the EAT-Lancet Commission’s report on ‘Food in the Anthropocene’ (51) was criticised on the grounds of affordability and cultural (in)appropriateness (52). There have also been criticisms of the study’s lack of transparency and replicability (53). Whilst such criticisms might be said to reflect a partial misunderstanding of what was entailed in identifying a global ‘reference diet’ (the *typical* diet required for planetary and human heath rather than a *universal prescription* of what everyone should eat, irrespective of their circumstances), it highlights the importance of cultural context and economic feasibility in formulating dietary recommendations. This is also recognised in the FAO’s guiding principles on the development of healthy and environmentally sustainable diets, which recognise, amongst a long list of other criteria, that they should be ‘built on and respect local culture, culinary practices, knowledge and consumption patterns, and values on the way food is sourced, produced and consumed’ (54).

For the Nordic and Baltic countries, food insecurity is relevant in this context, even though, until recently, this has not been prioritised in academic research (55). Results from the FAO’s monitoring of trends and progress towards the Sustainable Development Goals show the prevalence of moderate or severe food insecurity as follows: Denmark, Norway and Sweden approximately 5%; Iceland, Finland and Estonia 7–8%; and Latvia and Lithuania 10–12% (56). Nordic studies have shown that food insecurity is linked to low income and single parent households (57), and recent research on food insecurity in Denmark has shown that households facing severe food budget restraint had a higher probability of eating an unhealthy diet (58). For Estonia, unaffordability (regarding the consumption of meat, fish or poultry more than three times per week) has been shown to be linked to educational level and unemployment (59), and for Lithuania, being mildly food insecure was a higher risk for women and people with low income, whilst the risk of being moderately or severely food insecure was not related to gender but to the number of children in households and to levels of social capital (60).

Good evidence is also available on which to base more culturally appropriate nutritional advice. This includes research on the extent of ethnic differences in dietary intake in the Nordic countries. See, for example, Halkier and Jensen’s work on how Pakistani Danes incorporate nutritional advice into their dietary decisions (61); Brembeck and Fuentes’ work on the use of processed baby food amongst an ethnically diverse group of mothers in Falköping in western Sweden (62, 63); and Nielsen’s work on dietary advice and practices amongst ethnic minority and Danish parents (64, 65).

There are clear variations in the transition to more sustainable food practices across the Nordic and Baltic countries, which suggest that nutritional policies need to be adapted to reflect these different circumstances. For example, Niva et al. show how variations in support for environmental policies, interest in cooking and socio-demographic factors account for variations in the popularity of ‘local’ food across the Nordic countries, including resistance to proposed reductions in meat consumption (66). Johansson et al. explore Nordic children’s changing foodscapes, including the continued prevalence of ‘unhealthy’ foods at festive occasions (such as cosy evenings and birthday parties) (67). Mincyte and Plath have examined changing foodways in Estonia, Latvia and Lithuania showing how ethnic, national and class boundaries have been maintained and transgressed through changes in diet (68). Blumberg and Mincyte show how the taste for ‘local’ food has been shaped by changing infrastructures from Soviet times through the market reforms of the 1990s to EU accession in 2004 (69), and how alternative food networks (AFNs) have prospered in Lithuania following the simplification of food safety and veterinary requirements for food products sold direct to consumers through farmers’ markets and other AFNs (70).

## Trade-offs and compromises

A recognition of the social and economic dimensions of food sustainability, from a food systems perspective, encourages an acknowledgement of the many trade-offs and compromises that are involved in dietary decisions where it is rarely possible to optimise all relevant criteria at once. Meah and Watson provide some good examples of the ethical dilemmas associated with food consumption, and how they are ‘negotiated into practice’ in people’s domestic provisioning choices (71). They show how consumers are frequently faced with trade-offs between convenience and cost, conscience and affordability, price and taste. They are particularly concerned about how poorer households feel unable to express their ethical preferences at the point of purchase (or, as one of their participants averred, how ‘morality is a privilege of the rich’). Meah and Watson document how consumers trade off their concern for distant producers, in the Global South, versus their desire to support ‘local’ farmers (in their home country) and how their preference for organic production may be compromised if it is transported by air over long distances compared to ‘local’ food that is intensively produced.

There has been a vigorous debate about the virtues of consuming ‘local’ food, questioning the idea of ‘food miles’ as a robust measure of sustainability. For example, whilst evidence suggests that food that is produced without artificial heating and imported by land and sea is likely to be more sustainable than food produced locally under artificial heat, ‘food miles’ remain high on many consumers’ climate agenda (72).

Similarly, Evans’ work on domestic food waste shows how even the most environmentally conscious consumers, strongly committed to the reduction of food waste, may still end up wasting food (73). Evans’ research traces how ordinary domestic practices configure food as waste, regardless of people’s personal motivations. He shows, for example, how the desire for fresh food can result in newly purchased food ‘pushing out’ previously purchased food, even when it is still in date. He shows how the aspiration for families to eat together runs up against the time constraints and dietary preferences of different family members leading to food being wasted, and how the desire for dietary variation can thwart well-intentioned efforts at large-scale batch-cooking, designed to reduce food waste. There is, in Evans’ research, little evidence of consumer profligacy characterised by a casual or careless disregard for food (74). Rather, food provokes a range of complex emotions (from guilt and anxiety to care and concern) and waste results from a constellation of ordinary domestic practices including routines of food provisioning, the infrastructures and institutions that support these practices, and the competing demands of family life (75, 76).

This body of work also shows how cultural constructions of taste, convenience, hygiene and risk all shape dietary decisions beyond the calculation of calories, minerals and carbohydrates that figure so prominently in nutritional advice. For some ethnographic insights into how ‘taste’ is culturally constructed in social as well as individual terms (77). Work has also focused on cultural constructions of ‘convenience’ food and its implications for policy and practice (78); and on the implications of everyday domestic practices for kitchen hygiene, including the risks of cross-contamination and the spread of foodborne disease in both a British (79) and a Norwegian context (80). Paying attention to the wider social and cultural context of food consumption and sustainability introduces a whole range of additional ethical and emotional considerations. For example, Garnett suggests that definitions of a sustainable diet need to consider the complex meanings of food, including pleasure, guilt, ritual, status, comfort, bribery and love (81).

Taking account of the social and economic dimensions of sustainability also focuses attention on the temporalities of food system change. It is, for example, relatively easy to measure short-term increases in public awareness of health campaigns but much harder to measure the longer-term impact of specific interventions on public health (noting that the impacts of dietary change have long lead-times and are subject to many intervening factors). The lack of long-term evaluation is a notorious weakness of many public health interventions, and similar criticisms apply to the outcome of sustainability initiatives (82, 83).

Similar questions can be raised about the durability of policy initiatives and whether dietary interventions have lasting impacts. There is no shortage of evidence on the social embeddedness of dietary practices, which make behaviour change initiatives so challenging, especially where systemic issues are addressed in a largely individualistic manner (84). Diets are widely acknowledged to be deeply embedded in the practices of everyday life, freighted with cultural and moral significance and entrenched within powerful institutions and economic interests – all of which render diets highly resistant to change. This would suggest that changes to dietary guidelines and advice are unlikely to be effective unless they are set within wider changes involving government, industry and civil society. For example, obesity policy is increasingly being recast in ways which question the role of individual agency and ‘informed choice’ (85), placing more emphasis on the wider ‘food environment’ including the encouragement of product reformulation, changes in portion size, restrictions on fast-food advertising to children and similar measures. There has been some work on this topic by the Norwegian public–private partnership for a healthier diet, which recommends actions on product reformulation, portion size and marketing measures (86). The food industry has also implemented a self-regulation scheme for responsible marketing practices towards children and youth (87). Evaluating the success of such measures in promoting healthier and more sustainable diets should be a priority for future research.

Finally, when the social context of food consumption is taken into account, attention is likely to focus on *meals* and other eating occasions rather than a more limited focus on individual *ingredients* and nutritional levels. This recognition is fundamental to the sociology of food and eating as discussed by Murcott and others (88). Murcott shows how conventional ideas about family meals, ethnic cuisines, cooking skills and convenience foods, eating out, food waste and packaging can be challenged by the kind of critical thinking that is inspired by a sociological perspective on these apparently mundane concerns. Food-based guidelines therefore need to address the consumption of foods *during specific meal occasions* as well as their composition in terms of specific nutrients.
